# *Mycobacterium tuberculosis* Invasion of the Human Lung: First Contact

**DOI:** 10.3389/fimmu.2018.01346

**Published:** 2018-06-12

**Authors:** Jeroen Maertzdorf, Mario Tönnies, Laura Lozza, Sandra Schommer-Leitner, Hans Mollenkopf, Torsten T. Bauer, Stefan H. E. Kaufmann

**Affiliations:** ^1^Max Planck Institute for Infection Biology, Berlin, Germany; ^2^Lungenklinik Heckeshorn, HELIOS Klinikum Emil von Behring, Berlin, Germany

**Keywords:** *Mycobacterium tuberculosis*, innate immunity, pulmonary infection, tissue-resident cells, host–pathogen interaction

## Abstract

Early immune responses to *Mycobacterium tuberculosis* (Mtb) invasion of the human lung play a decisive role in the outcome of infection, leading to either rapid clearance of the pathogen or stable infection. Despite their critical impact on health and disease, these early host–pathogen interactions at the primary site of infection are still poorly understood. *In vitro* studies cannot fully reflect the complexity of the lung architecture and its impact on host–pathogen interactions, while animal models have their own limitations. In this study, we have investigated the initial responses in human lung tissue explants to Mtb infection, focusing primarily on gene expression patterns in different tissue-resident cell types. As first cell types confronted with pathogens invading the lung, alveolar macrophages, and epithelial cells displayed rapid proinflammatory chemokine and cytokine responses to Mtb infection. Other tissue-resident innate cells like gamma/delta T cells, mucosal associated invariant T cells, and natural killer cells showed partially similar but weaker responses, with a high degree of variability across different donors. Finally, we investigated the responses of tissue-resident innate lymphoid cells to the inflammatory milieu induced by Mtb infection. Our infection model provides a unique approach toward host–pathogen interactions at the natural port of Mtb entry and site of its implantation, i.e., the human lung. Our data provide a first detailed insight into the early responses of different relevant pulmonary cells in the alveolar microenvironment to contact with Mtb. These results can form the basis for the identification of host markers that orchestrate early host defense and provide resistance or susceptibility to stable Mtb infection.

## Introduction

Pulmonary infections account for a staggering death toll worldwide, with tuberculosis (TB) impacting profoundly on morbidity and mortality (1). When an exposed individual inhales *Mycobacterium tuberculosis* (Mtb), the initial responses in the lung are considered to play a decisive role in the outcome of infection. While Mtb frequently establishes stable infection upon inhalation, increasing evidence suggests that in a substantial number of cases the bacteria are cleared. Such early clearance of the invading bacteria (2) relies on natural resistance mechanisms that eradicate inhaled Mtb without the need for an adaptive immune response. For example, in defined cohorts of health-care workers with close and repetitive contact to TB patients, a significant number of individuals showed no sign of adaptive immunity to Mtb (3, 4), suggesting early eradication of the bacteria before stable infection has been established.

Although innate responses of pulmonary cells to viruses and bacteria have been widely studied (5), interactions between these infectious agents and the various cell types in the human lung are still poorly understood. Generally, *in vitro* cell culture models usually focus on a single cell type, ignoring the complex cellular composition, as well as the architecture of human lung tissue (6). Primary responses of lung tissue to Mtb invasion have also been studied in experimental animal models, mostly in mice. Aside from species differences, however, the clean and semi-sterile conditions under which these animals are kept is way off from the real-life situation in which human beings are constantly exposed to dust, pollen, infectious agents and other airborne particles. Moreover, these models lack the biological heterogeneity and diversity in living conditions in human populations.

Investigating immunity against Mtb in the human lung may provide new insights into the mechanisms of early clearance and host resistance to infection in TB (2, 4, 5, 7). This could lead to identification of biomarkers of potential use for the development of efficacious vaccines (7). The main goal of our study was to analyze the primary responses of different relevant pulmonary resident cells to Mtb in their natural environment, i.e., the human lung, using fresh human lung tissue explants. Human lung *ex vivo* infection models have been used before to study host–pathogen interactions in human lung tissue (6). As far as we are aware, only one such infection model has been applied in the context of Mtb infection (8). Whereas this study mainly focused on the distribution of infected cell types and their morphologic changes, we investigated the biological responses of the various cell types in the human lung to Mtb exposure.

Here, we provide a comprehensive data set of early responses of the different cell types comprising the human lung architecture and various tissue-resident innate lymphocytes (9). Initial cellular responses, which were mainly analyzed on the gene expression level, were dominated by activation and increased proinflammatory chemokine responses in myeloid cells. These responses were most prominent in the cell types aligning the alveolar space which are in direct contact with invading pathogens, i.e., alveolar macrophages (AM) and epithelial cells. Finally, we link these primary proinflammatory signals with responses of tissue-resident innate lymphoid cells (ILCs) in the lung.

## Materials and Methods

### Human Lung Tissue Collection and Ethics

Lung tissue explants were obtained from patients at a thoracic center in Berlin, undergoing lung surgery for clinical reasons unrelated to pulmonary infections. The protocol was approved by the Charité ethics committee Berlin (project number EA2/012/13). All subjects gave written informed consent in accordance with the Declaration of Helsinki.

Fresh healthy parts of resected lung tissue were kept and transported in saline and cut into smaller sections (approx. 3–5 mm in size) and either used immediately for infection experiments or left overnight in RPMI medium (containing 2 mM l-glutamine and 5% human serum without antibiotics). The size of each tissue section was visually estimated, distributing smaller and larger sections equally between the different experimental conditions. Tissue donors had no other pulmonary infections or major inflammation at time of lung resection. We had no information on age, gender, nor on potential asthma/allergy or steroid use of the participants. Due to sample limitations and logistical restrictions, results described in this manuscript are derived from three separate batches of donor material: data on whole tissue responses are from 11 individual donors; RNAseq analysis on the major innate lung cell types was performed on tissue samples from eight donors; for RNAseq on ILCs, 8 donor tissues were deployed.

### Mycobacterial Strains and Tissue Infection

*Mycobacterium tuberculosis* strains (H37Rv, H37Ra and Beijing) were cultured in Middlebrook 7H9 broth (BD, Heidelberg, Germany) containing 0.05% glycerol and Tween-80, and enriched with 10% ADC (BD). H37Rv is a well-characterized virulent Mtb strain, most widely used in laboratory work; H37Ra is an avirulent strain of Mtb due to a mutation in the transcriptional regulator PhoP (10); the Beijing strain is a clinical isolate of Mtb with increased virulence which has spread globally (11). The three different Mtb strains were used for strain-specific responses by microarray. Other infection studies used H37Rv only. Colony-forming units (CFUs) in tissue sections were counted by lysing cells in 0.1% Triton-X and plating serial dilutions onto 7H11 Middlebrook agar plates.

For infections, tissue sections were briefly blotted onto Whatman filter to remove excess fluid and subsequently submerged in a bacterial suspension (5 × 10^6^ CFU in 2 ml medium) for 30 min to allow bacteria to invade the tissue. Afterward, tissue sections were gently rinsed to remove bacteria adhered to the outside surface. CFU counts were performed on three separate tissue sections per time point and donor to compensate for differences in size of the individual tissue sections. Samples for direct whole tissue gene expression analyses or cell preparations for cell sorting were taken 20–24 h after infection, and treated lung pieces from each donor sample were pooled for cell preparation. Cellular viability was not assessed in between. For all experiments, uninfected pieces of tissue from the same donor were processed in parallel to infected ones as matched uninfected controls.

### Cytokine/Chemokine Measurements

Cytokine and chemokine levels in supernatants from infected lung tissue sections were analyzed on a Luminex 100/200 machine, using the human group I (27-plex) and Th17 Bio-Plex assays (Bio-Rad) according to the manufacturer’s instructions. For each donor and time point, supernatants from three wells containing pieces of infected lung tissue were pooled to average out differences in tissue size.

### Cell Preparation and FACS Sorting

Single cell suspensions from infected tissue were prepared by cutting sections into smaller fractions and incubating them for 20 min in a PBS solution containing collagenase A (1 mg/ml), dispase II (1 mg/ml), and DNase I (0.5 mg/ml) (Sigma-Aldrich). The digested tissue pieces were subsequently pressed through a 40 µm cell strainer, followed by two washing steps.

For FACS staining and cell sorting, the following fluorescently labeled antibody panels were used:

Staining of AM, epithelial (epi) and endothelial (endo) cells: CD3-PerCP/Cy5.5 (clone), CD31-PB (clone WM59), CD45-AF700 (clone HI30), CD64-APC/Cy7 (clone 10.1), CD146-PE (clone P1H12), CD324-APC (clone 67A4), and CD326-FITC (clone 9C4).
For innate gamma/delta (γ/δ) T cells, mucosal associated invariant T (MAIT) cells, natural killer (NK) and NK T (NKT) cells: CD3-BV510 (clone OKT3), CD45-Af700 (clone HI30), CD56-APC (clone HCD56), CD69-PB (clone FN50), CD161-PE (clone HP-3G10), TCR pan γ/δ-FITC (clone IMMU510), and TCRVα7.2-Pe/Cy7 (clone 3C10).
Innate lymphoid cells: CD3-PE/Dazzle 594 (clone UCHT1), CD45-AF700 (clone H130), CD94-FITC (clone DX22), CD117-BV510 (clone 10402), CD127-BV421 (clone A016D5), CD161-Pe/Cy7 (clone HP-3G10), CRTH2-PE (clone BM16), and NKp44-AF647 (clone P44-8).
Lineage markers: CD11c-PerCP/Cy5.5 (clone BU15), CD14-PerCP/Cy5.5 (clone HCD14), CD19-PerCP/Cy5.5 (clone H1B19), CD123-PerCP/Cy5.5 (clone 6H6), and FcɛR1A-PerCP/Cy5.5 (clone AER-37).

All antibodies were purchased from BioLegend, except for TCR pan γ/δ-FITC (Beckman Coulter). Target cells were sorted directly into lysis buffer on a BD FACSAria II machine, with subsequent RNA extraction using the Qiagen RNeasy plus micro kit.

### Microarray Analysis

Lung tissue was disrupted in Trizol for RNA extraction. RNA was labeled with the Fluorescent Linear Amplification Kit (Agilent Technologies) according to the manufacturer’s instructions and hybridized to whole-genome 4 × 44k human expression arrays (Agilent Technologies). Raw data from scanned arrays were generated with Feature Extraction software (version 10.5.1, Agilent Technologies).

### RNAseq

For gene expression analyses of sorted cell subsets, RNAseq libraries were generated as follows: messenger (m)RNA was reverse transcribed and pre-amplified using the SMART-Seq v4 ultra low input RNA kit (Takara Clontech), followed by library generation using a Nextera XT DNA library prep kit (Illumina). Sequencing of the libraries was done on an Illumina HiSeq 1500 machine.

### Analysis

Microarray data were analyzed with R package limma (12) for assessment of differential expression; gene module enrichment analysis was done with R package tmod (version 0.36)^1^ (J. Weiner). Filtered RNAseq reads were aligned to the human genome using the STAR aligner (version 2.5.3a)^2^ (A. Dobin), read counting was done with the tool htseq-count of the Python package HTSeq (13), and expression analysis was performed with the R package EdgeR (14).

## Results

### Tissue Infections

**Figure 1:**
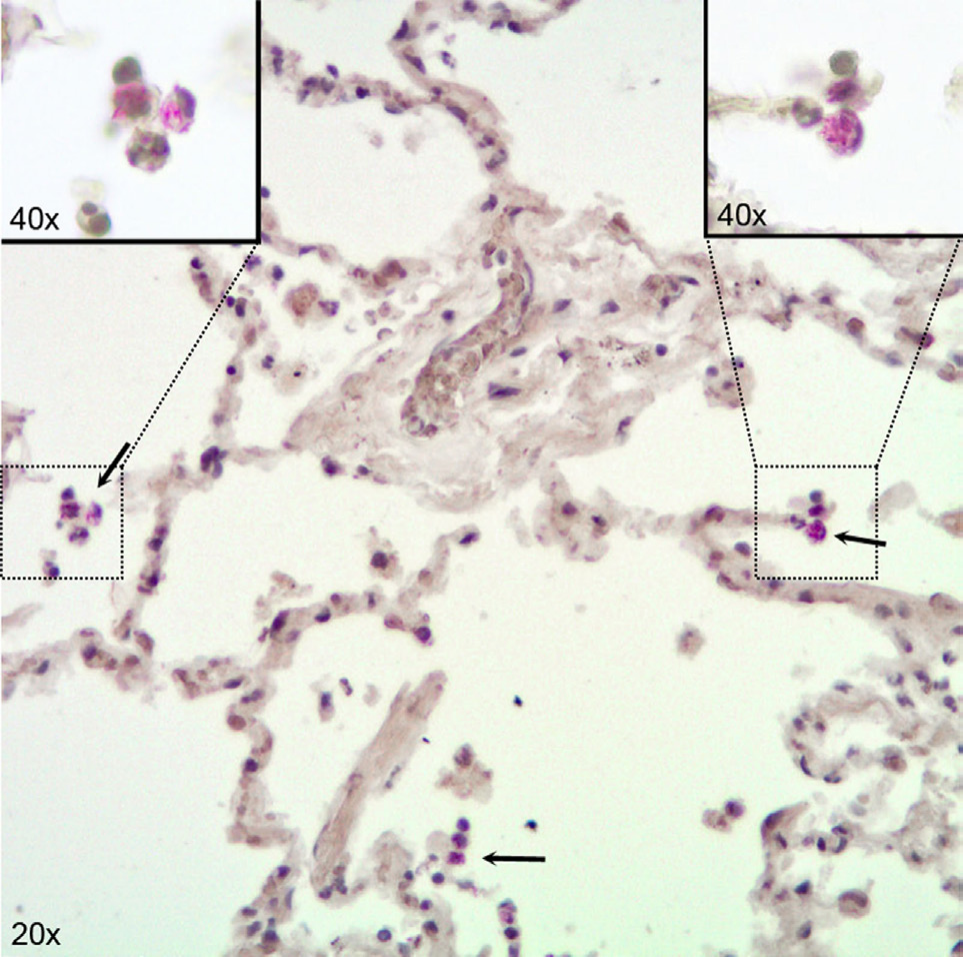
Immunohistochemical visualization of *Mycobacterium tuberculosis* in alveolar macrophages (AM) in infected human lung tissue. Lung tissue formalin fixed 24 h after infection with H37Rv. Stained for acid fast bacilli by the Ellis and Zabrowarny technique (purple). Arrows point to AM containing bacilli, with insets showing higher magnification of selected cells.

In our infection model, we detected substantial numbers of viable bacteria inside infected lung tissue, both by basic histological examination and by CFU counts on infected tissue. In histological sections, we observed most bacteria inside AM and some bacteria free in the alveolar space (Figure 1). Using this infection model, several hundred thousands of bacteria entered the lung tissue, as estimated from CFU counts on infected pieces right after infection and rinsing the tissue sections to remove bacteria adhered to the outside (Figure S1 in Supplementary Material). Roughly 10% of the initial number of viable bacteria survived the first day after infection and CFU counts virtually remained unchanged afterward (Figure S1 in Supplementary Material). CFU counts obtained from infected tissue were highly variable, and counting was discontinued because of too many unreliable readings. We instead focused on biological responses of different relevant cell types in human lung tissue. First, we analyzed whole tissue responses with emphasis on the main cells that make up the basic lung tissue structure which are in direct contact with invading pathogens, i.e., AM and epithelial cells, and also included endothelial cells in our analyses.

### Whole Tissue Responses

**Figure 2:**
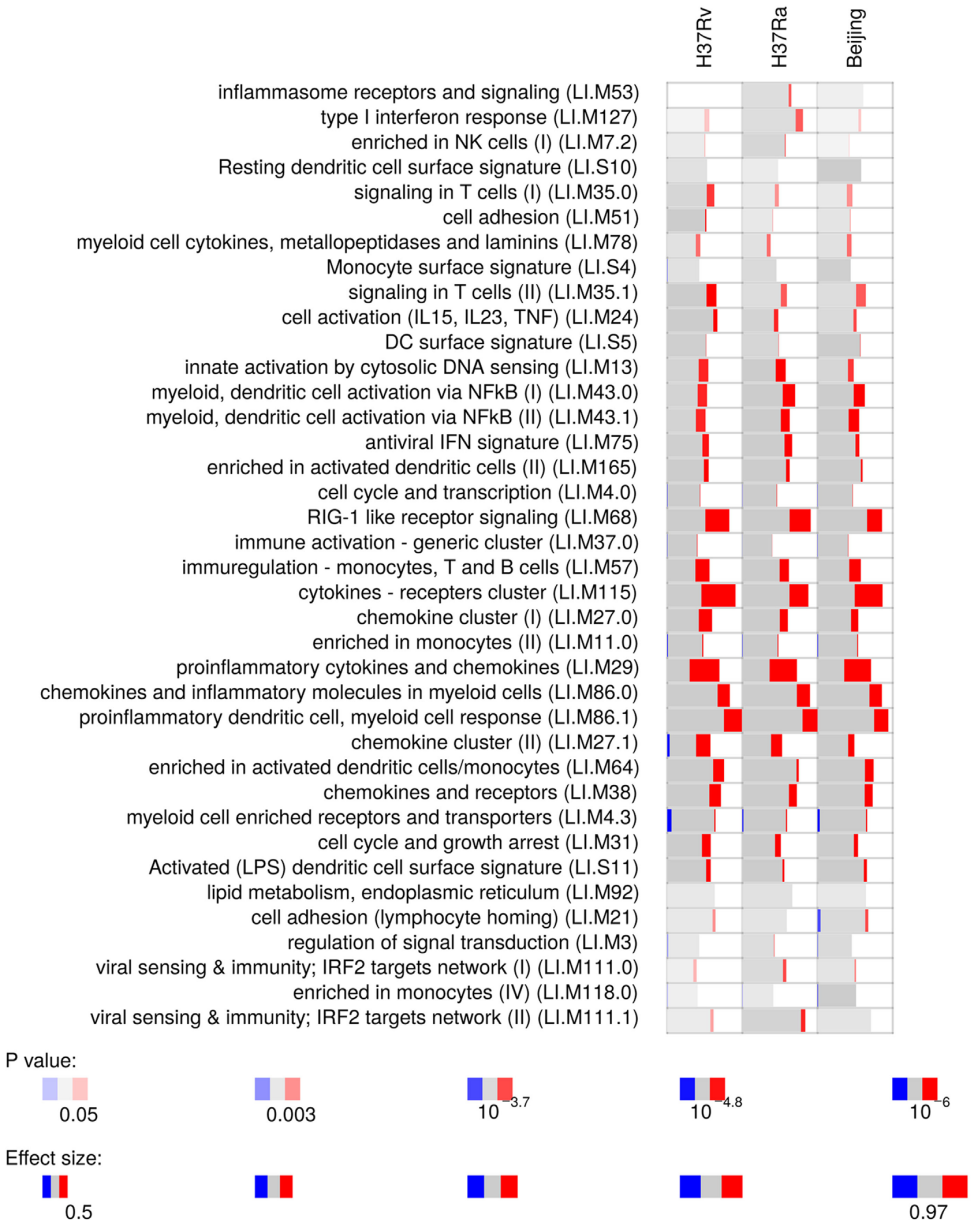
Gene module enrichment of lung tissue infected with different strains of *Mycobacterium tuberculosis*, based on microarray gene expression analyzed with R package tmod. Red indicates the proportion of significantly upregulated genes in each module given on the *y*-axis.

Microarray analysis on whole infected tissue revealed a strong proinflammatory response of myeloid cells (Figure 2). Infected tissue responded with marked induction of IL1-β, both at the gene expression and protein level (Figure S2 in Supplementary Material). We did not observe any Mtb strain-specific differences in responses induced by the three Mtb strains used. These data suggest that virulence of Mtb has minor impact on prompt proinflammatory responses on myeloid cells. Hence, subsequent tissue infections were done with the virulent Mtb H37Rv strain only.

**Figure 3:**
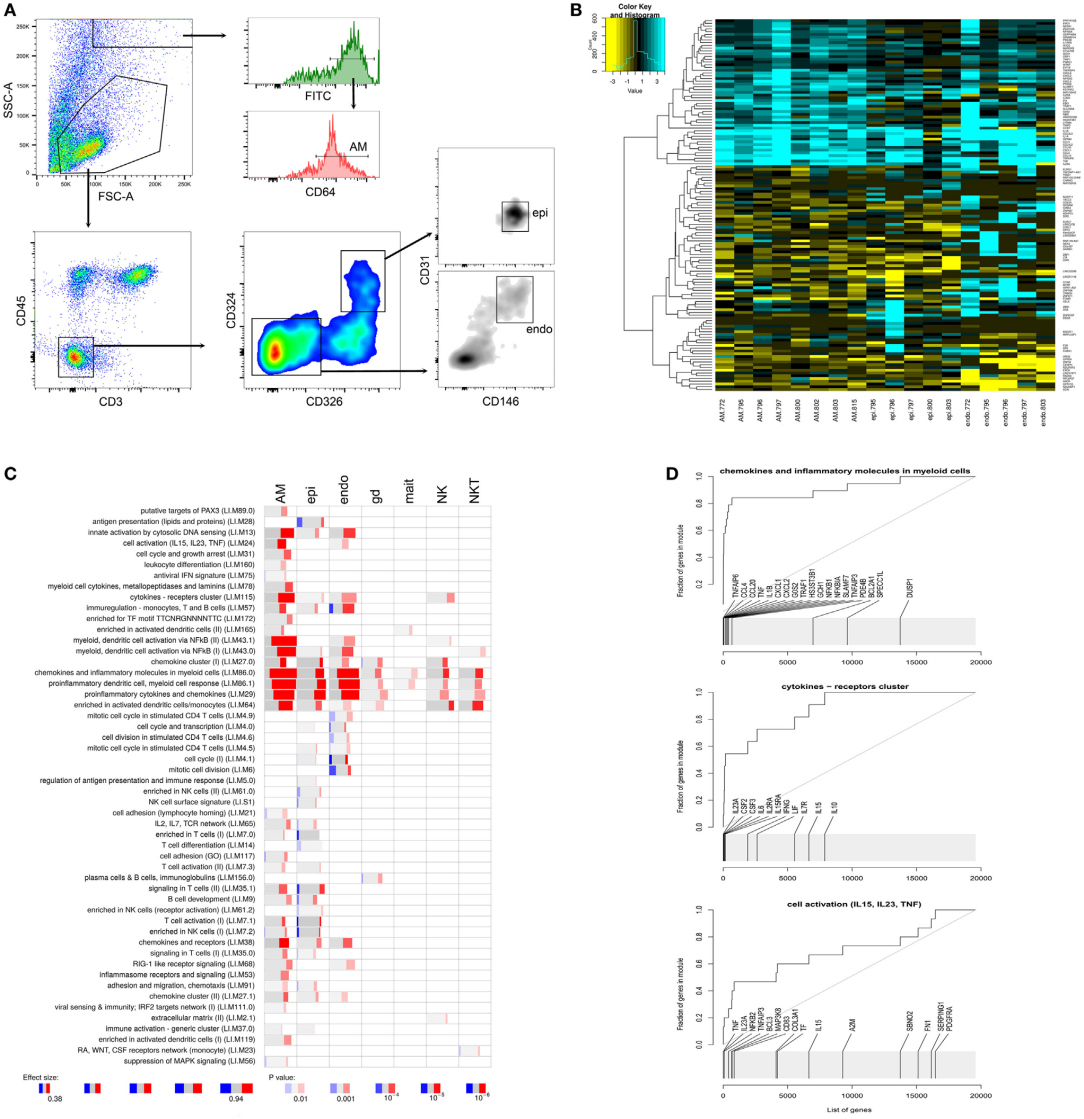
Analysis of distinct pulmonary cells. **(A)** Gating strategy for alveolar macrophages, epithelial cells (epi), and endothelial cells (endo). **(B)** Heatmap displaying log2 fold differences between cells from infected and uninfected for each donor. Shown are unique transcripts from a combined list of the top 50 differentially expressed transcripts for each cell type. **(C)** tmod gene module enrichments in innate pulmonary cells from infected versus uninfected tissue. gd, γ/δ T cells; mait, mucosal associated invariant T (MAIT) cells; natural killer (NK) and natural killer T (NKT) cells. **(D)** tmod evidence plots showing ranking of individual genes from three prominently enriched gene modules in panel **(C)**. A high resolution version of this figure is supplied as Figure S5 in Supplementary Material.

To identify the cell types responsible for these proinflammatory responses, we FACS sorted AM, epithelial and endothelial cells (Figure 3A), the latter two as the main cell types that make up the tissue architecture of the lung. We then analyzed gene expression within the different cell types using RNAseq. In parallel, we also isolated other innate lung resident cells from the same donor tissues: γ/δ T cells, MAIT cells, NK cells, and NKT cells. (See Figure S3 in Supplementary Material for gating strategy of the latter cell types.)

### Transcriptional Responses in Distinct Tissue Cells

When analyzing individual cell types from infected lung tissue sections, we observed a strong induction of proinflammatory chemokines and cytokines in myeloid cells, primarily in AM, and to a lesser extent in epithelial and endothelial cells (Figures 3B,C). The other innate tissue-resident cells under investigation, i.e., γ/δ T cells, MAIT cells, NK cells, and NKT cells displayed less pronounced enrichments of these proinflammatory signatures (Figure 3C) but still showed increased expression of some of the key proinflammatory markers (Figure S3B in Supplementary Material).

The most prominently induced genes included *IL1B* and IL8 (*CXCL8*), which were upregulated in all isolated cell types after infection of the lung tissue (Figure S4 in Supplementary Material). Gene module enrichment analysis also indicated signaling in T cells and activation of cells through IL15, IL23, and tumor necrosis factor (TNF), which are important modulators of inflammatory responses (Figures 3C,D). IL23A can act in conjunction with IL17 causing detrimental inflammation (15). Although the elevated gene expression levels for *IL17* in infected tissue were not statistically different, most donors displayed increased secretion of IL17 cytokines (Figure S2 in Supplementary Material).

**Figure 4:**
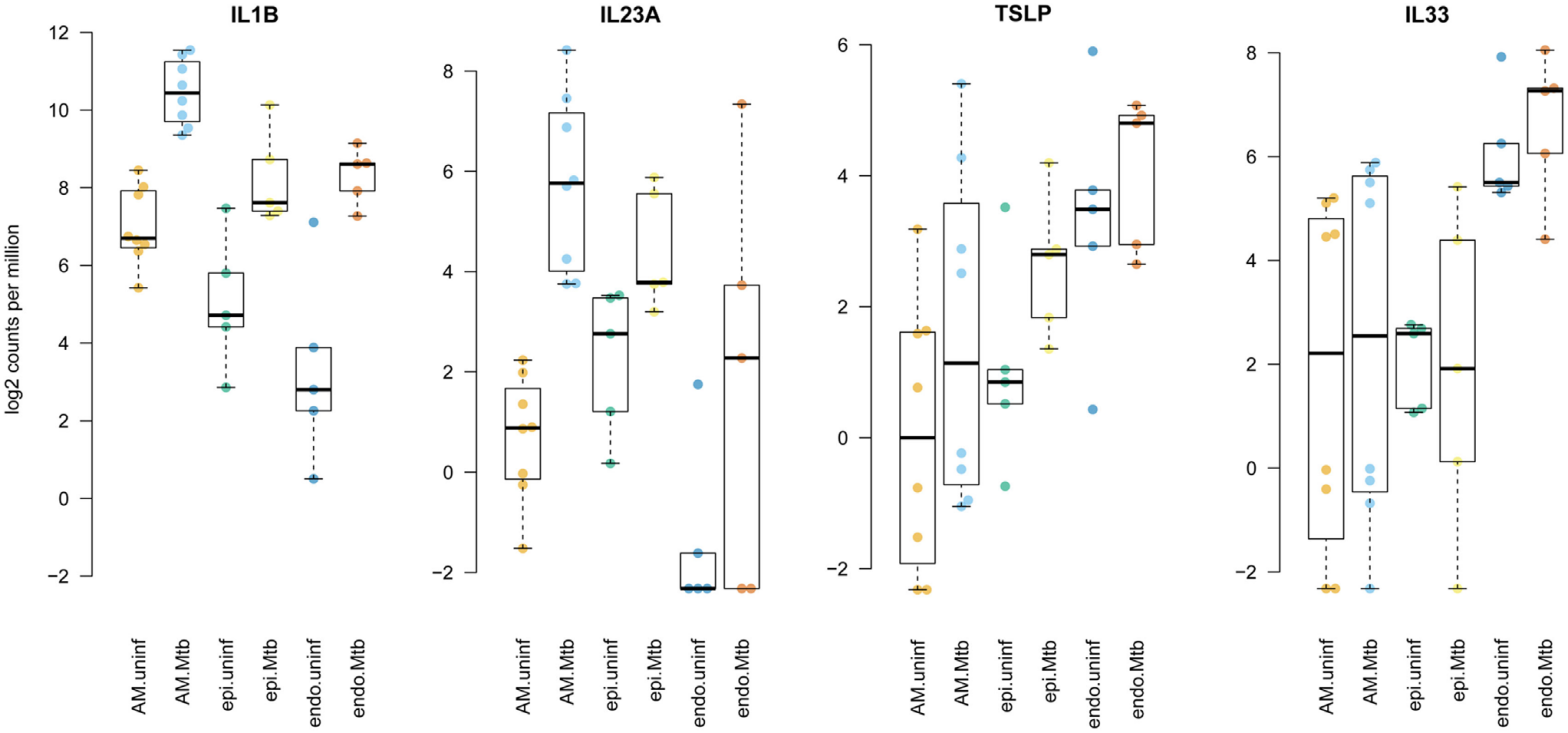
Enhanced expression of innate lymphoid cell stimulating cytokines by distinct tissue-resident cells upon stimulation with *Mycobacterium tuberculosi*s (Mtb). Plotted are log2 read counts per million sequenced reads from isolated cells for each donor tissue sample. Boxplots show median plus first and third quartile; whiskers extend to the most outer data point within 1.5 times the interquartile range.

These responses in innate lung tissue cells included strong activation of proinflammatory molecules that act on ILCs. For example, IL1-β (gene *IL1B*) and IL23, which were markedly induced by Mtb in our infection model (Figure S4 in Supplementary Material), are known to induce group 3 ILC (ILC3) (16, 17); IL15 among others activates ILC1 cells, and IL1-β, thymic stromal lymphopoietin (TSLP) and IL33 are prominent activators of ILC2 cells (17–20). *TSLP* expression was upregulated in AM and epithelial cells after Mtb infection, whereas *IL33* was mainly produced by endothelial cells and not affected by Mtb stimulation (Figure 4). Increased secretion of TSLP in infected tissue was also clearly observed at the protein level (Figure S2 in Supplementary Material). Since ILCs are key regulators of homeostasis and conductors of tissue immunity (17, 21), we investigated their behavior in human lung tissue upon Mtb invasion.

### ILC Responses

**Figure 5:**
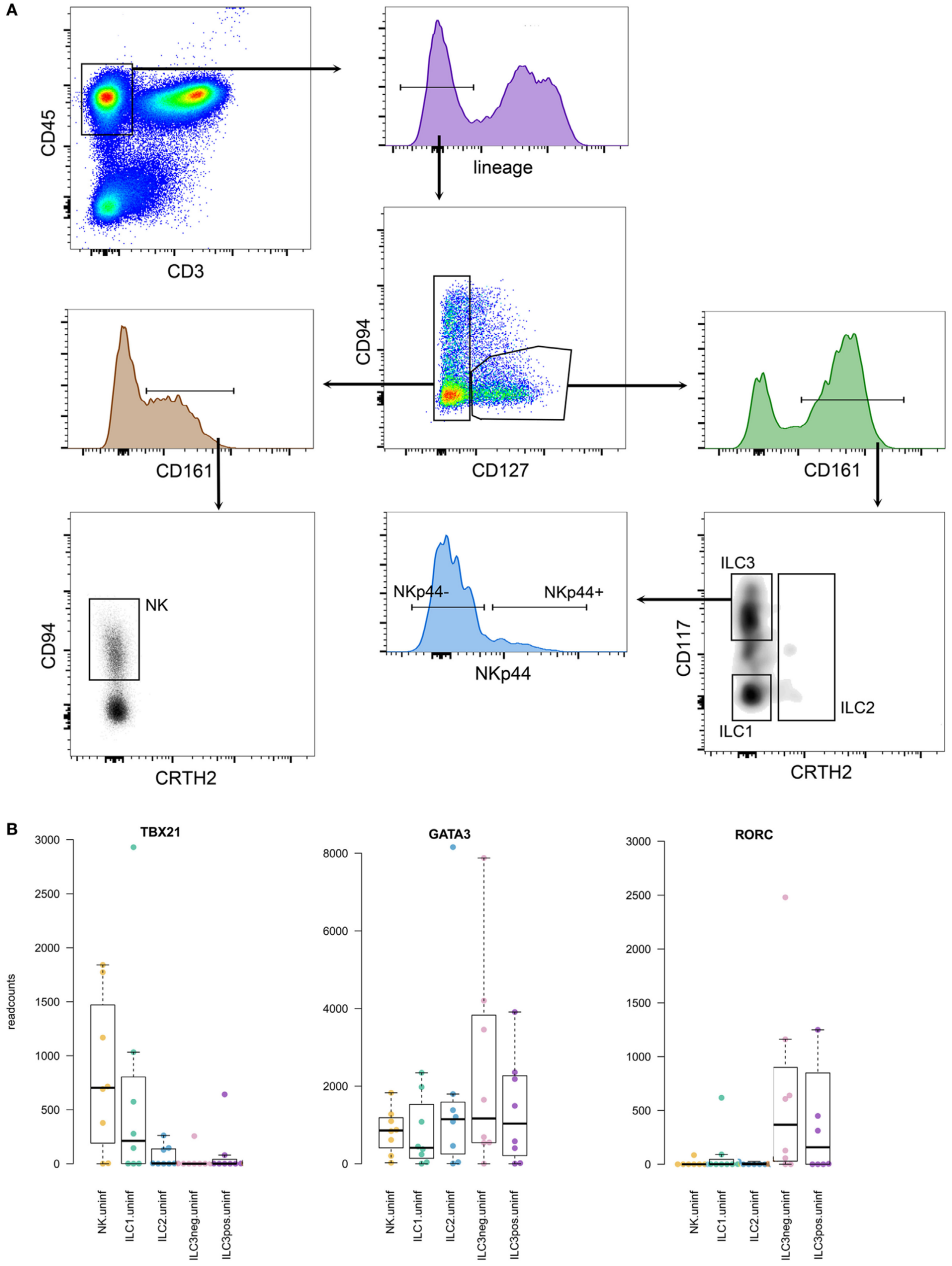
**(A)** Gating strategy for innate lymphoid cells (ILCs), gated on lymphocytes in forward-side scatter. **(B)** Expression levels of prototypic transcription factors in the different ILCs analyzed.

Principally, different groups of ILCs are characterized by the specific expression of distinct transcription factors. Since our RNAseq analyses required high quality RNA, we could not stain cells for these particular transcription factors (for which the cells would need to be fixed resulting in RNA degradation), and relied on surface markers only. Our ILC gating strategy was based on phenotypic surface markers as described by Juelke and Romagnani and Hazenberg and Spits (22, 23) and shown in Figure 5. As a reference, we also generated expression profiles from the whole single cell suspensions used for FACS sorting of the different ILCs. The number of isolated ILCs using our strict gating strategy was usually low, and the composition of the different ILCs was highly variable between donors. For bonafide ILC1, several hundreds to thousands of cells were sorted, while for ILC2 and ILC3, the number of identified cells ranged from a few dozen to a few 100. Group 3 ILCs were sorted into NKp44 negative and positive sub fractions. NK cells belonging to the group 1 ILCs were also isolated based on their CD127-negative phenotype. To verify that our gating strategy targeted the intended cells, we verified expression of key transcription factors frequently used to characterize different groups of ILCs (Figure 5). Transcription factor T-bet (gene *TBX21*) was mainly expressed in group 1 ILCs. *GATA3* expression was found in most sorted ILC fractions, while RORγt (gene *RORC*) expression was restricted to group 3 ILCs, as expected (23).

**Figure 6:**
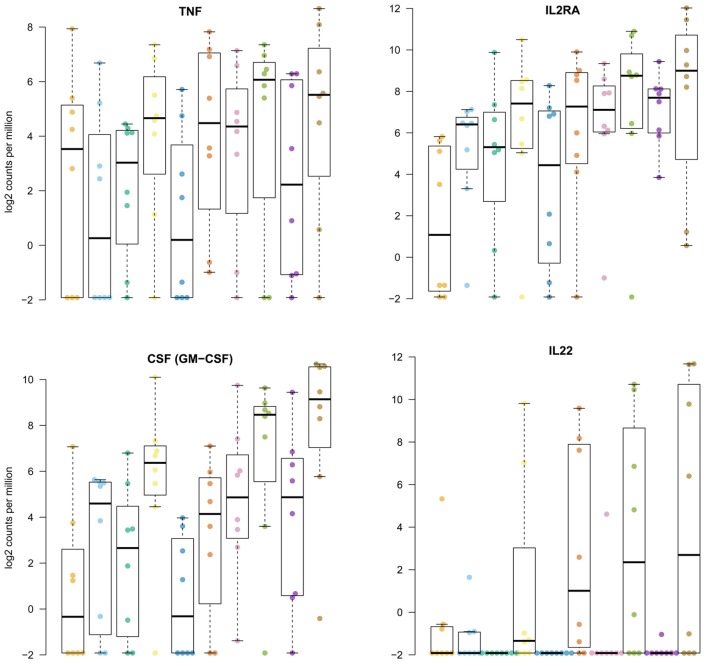

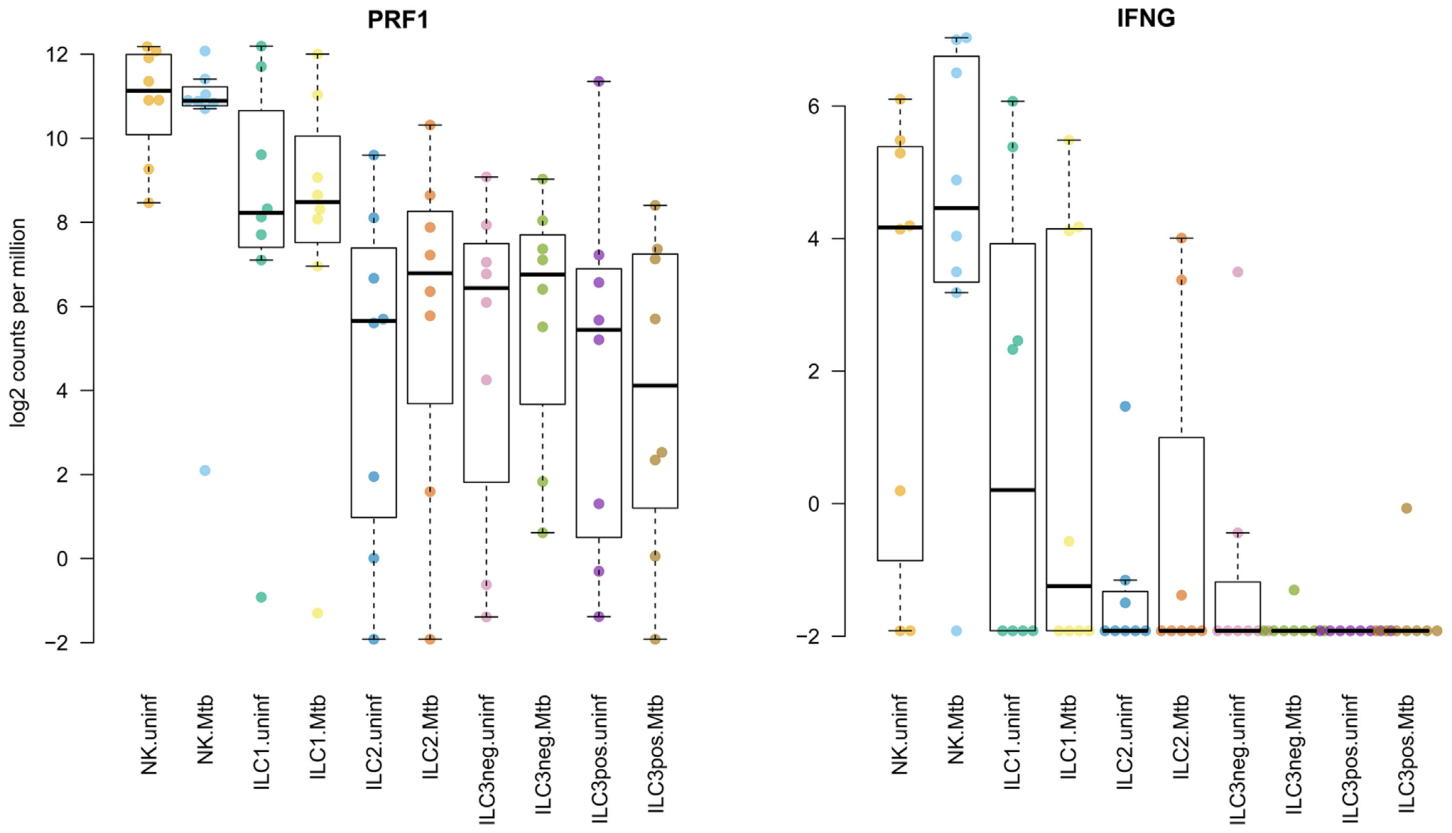
Induction of regulatory cytokines in innate lymphoid cells in human lung tissue upon *Mycobacterium tuberculosis* (Mtb) stimulation. Plotted are log2 read counts per million sequenced reads from isolated cells for each donor tissue sample. Boxplots show median plus first and third quartile; whiskers extend to the most outer data point within 1.5 times the interquartile range.

Reflecting the variability between tissue samples observed during FACS sorting, the RNAseq data revealed highly variable levels of gene expression between donors (Figures 5 and 6). Similarly, induction of gene expression in the Mtb stimulated fractions were sometimes seen in only part of the donors. Although hundreds of genes were differentially expressed in human lung tissue ILCs, gene set enrichment did not reveal distinct biological signatures in response to Mtb. Hence, we primarily focused on regulatory cytokines and other molecules that have previously been found to be expressed by ILCs upon stimulation. TNF production, which is usually associated with group 1 ILCs and promotes macrophage activation (17, 18) was elevated in ILC1 and ILC2 from Mtb infected lung tissue (Figure 6). CD25 (*IL2RA*) was upregulated primarily in group 1 and 2 ILCs (Figure 6), reflecting activation of these cells upon Mtb infection. The main response factors produced by group 3 ILCs include GM-CSF (gene *CSF2*) and IL22, which stimulate phagocytosis and production of antimicrobial peptides (17, 18). These factors were also observed in our study, revealing upregulation of gene expression in both group 3 ILC subsets (Figure 6). The other groups of ILCs showed a similar response to Mtb. *IL22* gene expression was absent from uninfected tissue. Upon Mtb infection, several donors responded with strong induction of *IL22* while others did not, illustrating the heterogeneity in tissue responses between donors.

Being an effector molecule of group 1 ILC NK cells, perforin (gene *PRF1*) was consistently highly expressed in group 1 ILCs, but its expression was not affected by Mtb infection. Group 1 ILC NK cells also displayed induction of interferon-γ (gene *IFNG*) in response to Mtb. For several donors, unstimulated lung tissue already showed significant levels of *IFNG* expression (Figure 6). Several cytokines that have been described as effector markers for group 2 ILCs, including IL4, IL5, and IL13 were not detected or only slightly expressed in some samples. The same was observed for IL17 gene expression in group 3 ILCs.

## Discussion

Innate resistance to Mtb exposure is strongly indicated by observations that groups of health-care workers who, despite prolonged close contacts with TB patients, neither develop TB disease nor adaptive immune responses to Mtb (3). Apparently, these individuals can clear the invading pathogen at an early stage of encounter (2) and hence prevent stable Mtb infection.

Early pulmonary defense to Mtb has partially been studied in animal models. In the rabbit model, for example, the outcome of Mtb infection depends, at least in part, on the type of early innate immune response (24). In the mouse model, pre-stimulation of innate immunity was found to have a protective effect on pulmonary infections (25). In contrast to laboratory animals being kept under clean and semi-sterile housing conditions, humans are constantly exposed to airborne particles, environmental microbes, and pathogens. While excessive exposure (e.g., through air pollution) is detrimental, continuous stimulation in real-life situations can train pulmonary cells for accelerated responses to invading bacteria (4, 25, 26).

Alveolar epithelial cells and AM are the main cell types in the alveolar space which are in direct contact with inhaled pathogens. The alveoli are lined by epithelial cells, which form the tissue surface in contact with the outside environment and perform an important barrier function against invading pathogens (27). While their primary functions are gas exchange and maintenance of pulmonary homeostasis, they also play a critical role in balancing immunity in the lung between resistance to infection and tissue damage (28). Alveolar epithelial cells exert their major role in the innate response to bacterial invasion through the secretion of antimicrobial peptides and proinflammatory factors (29). Resident AM in the alveolar space provide another first barrier by clearing airborne particles and pathogens. In response to bacterial invasion, they secrete proinflammatory mediators to recruit neutrophils and monocytes, and they communicate with epithelial cells to fine-tune pulmonary immune responses (30).

The role of endothelial cells in pulmonary immunity is less well defined, although they do play a key role in the pathology of asthma (31). Our data indicate that they also participate in innate immune responses to Mtb invasion in the lung, similar to epithelial cell function. Thus, endothelial cells may play a more prominent role in lung immunity than recognized so far. Another more central component of the cellular architecture of human lung tissue is pulmonary fibroblasts. Due to their sticky nature, we failed to consistently isolate these cells from tissue samples. Together with other tissue-resident innate lymphocytes (γ/δ, MAIT, NK, and NKT cells) (9), these pulmonary cells orchestrate the inflammatory response upon invasion of Mtb in human lung tissue.

Hocke et al. (6) recently reviewed the use of lung explant models for various bacterial and viral infections. For Mtb, human lung explants have thus far only been used to study cellular infection and morphology (8). We realize that in infection models such as ours, the abundance of invading bacteria is probably much higher than in real-life situations. However, to study the initial transcriptional responses of tissue and individual pulmonary cells to infection, a substantial proportion of cells need to come into contact with the bacteria to achieve a measurable signal above basic expression levels. An other limitation using lung explants is that the absence of cellular recruitment to the site of infection. We therefore exclusively focus here on the early response of tissue-resident cells in the first 24 h after infection.

Early responses to Mtb involved profound induction of proinflammatory cytokines and chemokines by myeloid cells. Such innate cytokines play a critical role in controlling initial infection and in regulating responses to restrict tissue damage (32). Proinflammatory factors induced include IL1-β and IL23, which both play a crucial role in the priming of adaptive immune responses (32). IL1 signaling is an important mediator in host resistance to Mtb and IL1-β in addition regulates functions of ILCs during human pulmonary inflammation (19, 20). IL23 maintains the inflammatory response to Mtb in mononuclear cells (32) and, in conjunction with IL17, can cause detrimental inflammation as it occurs in the so-called “Koch reaction” (33). Although we did not observe significant induction of IL17 gene expression, cytokine levels in supernatants of infected tissue were slightly increased in several donors. Gene expression of TSLP varied profoundly between donors; yet, induction by Mtb was consistently observed, particularly on the protein level. TSLP is an IL-7-like immunomodulatory cytokine which regulates lung homeostasis and barrier functions during inflammation (34–36). As a common feature, these innate mediators regulate and instruct ILCs.

Innate lymphoid cells are important regulators of immunity, with distinct roles in tissue homeostasis and inflammation (21, 37). Their functions mirror those of canonical T cells, but ILCs react more swiftly and in an antigen independent way (17). Whereas our current knowledge on ILCs is mostly derived from experimental mouse models and human peripheral blood cell studies, data on human lung ILCs are limited (22, 23, 37). Accordingly, the phenotypes of human ILCs remain incompletely understood (22, 23). Principally, group 1 ILCs promote innate responses to intracellular pathogens, group 2 ILCs respond to signals of tissue damage, and group 3 ILCs are primarily induced by extracellular microbes and fungi (17, 18, 21). In the human lung, ILC2s appear to be involved in pathology and inflammation, stimulated by increased TSLP expression in the tissue (22). Several typical responses of the different ILC subsets were induced by Mtb in our human lung tissue infection model. Thus, we observed differential expression in ILCs, without a distinct biological enrichment, likely due to the large variation in responses between different tissue donors and the restricted observation period of <24 h. By reducing the inoculum of Mtb, tissue integrity in this model can be maintained for several days, which may facilitate analysis of delayed ILC responses. On the other hand, human ILCs are also known to display a high level of plasticity, expressing diverse phenotypes in different microenvironments (16, 19, 20, 38, 39). This substantially complicates the identification of the distinct ILC subsets in the diverse donor samples in our study.

Innate immunity is crucial for host defense and resistance to Mtb infection. Early signals of inflammation by pulmonary cells that come in direct contact with the pathogen are paralleled by activation of other tissue-resident leukocytes. Together, they orchestrate inflammation to protect against infection and preserve tissue integrity (9, 21). With the data described here, we hope to set the basis for the identification of host markers that orchestrate early host defense and provide resistance to Mtb infection. A solid understanding of the innate defense responses in the human lung following Mtb invasion could pave the way for novel vaccines and immunomodulatory intervention measures (4, 40).

## Data Availability

All gene expression data generated and analyzed in this study are available in Gene Expression Omnibus (GEO). Whole tissue gene expression data are available under accession number GSE114911. RNAseq data on isolated cells under numbers GSE112483 (innate cell types) and GSE112482 (ILC data set).

## Ethics Statement

Lung tissue explants were obtained from patients at a thoracic center in Berlin, undergoing lung surgery for clinical reasons unrelated to pulmonary infections. The protocol was approved by the Charité ethics committee Berlin (project number EA2/012/13). All subjects gave written informed consent in accordance with the Declaration of Helsinki.

## Author Contributions

Designed research: JM, TB, and SK. Performed research: JM, MT, LL, SS-L, and HM. Analyzed the data: JM, LL, SS-L, and HM. Wrote the paper: JM and SK.

## Conflict of Interest Statement

The authors declare that the research was conducted in the absence of any commercial or financial relationships that could be construed as a potential conflict of interest.

## Footnotes

^1^https://CRAN.R-project.org/package=tmod.

^2^https://github.com/alexdobin/STAR/releases.

## Supplementary Material

Figure S1 Bacterial load in lung tissue sections at different days after infection. The number of viable bacteria is shown as colony-forming unit (CFU) per million in the inoculum, since infectious dose was not identical for all donor samples. Lines indicate bacterial counts in tissues from five individual donors.

Figure S2 Induction of IL1B, IL17, and thymic stromal lymphopoietin (TSLP) by human lung tissue upon infection with *Mycobacterium tuberculosis* (Mtb). **(A)** Normalized log2 gene expression values in uninfected and Mtb infected tissue sections. Boxplots show median plus first and third quartile; whiskers extend to the most outer data point within 1.5 times the interquartile range. **(B)** Cytokine levels in supernatants from uninfected and Mtb (H37Rv) infected tissue sections. Cytokine levels are mean values from three individual tissue sections per donor with lines connecting values from uninfected and infected samples from each donor.

Figure S3 **(A)** Gating strategy for innate tissue-resident cells, gated on lymphocytes in forward-side scatter. **(B)** Heatmap showing log2 fold changes of top differentially expressed genes for each donor and in all analyzed innate cells. Genes shown are unique transcripts from a combined list of the top 50 differentially expressed genes in each cell type.

Figure S4 Expression and induction of proinflammatory chemokine genes upon *Mycobacterium tuberculosis* infection of human lung tissue. Plotted are log2 read counts per million sequenced reads from isolated cells for each donor tissue sample. Boxplots show median plus first and third quartile; whiskers extend to the most outer data point within 1.5 times the interquartile range. Each dot represents expression levels from an individual donor.

Figure S5 High resolution version of Figure 3.
