# Supplementary figures and images for: Mycobacterium tuberculosis Invasion of the Human Lung: First Contact
Source: Front Immunol. 2018 Jun 12;9:1346. doi: 10.3389/fimmu.2018.01346 (PMC6022014; doi:10.3389/fimmu.2018.01346)

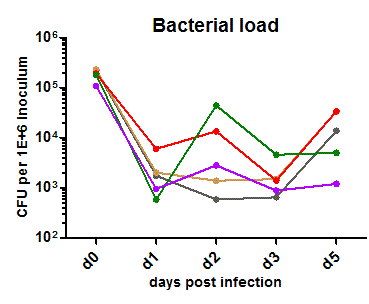

Supplement: Figure S1 — Bacterial load in lung tissue sections at different days after infection. The number of viable bacteria is shown as colony-forming unit (CFU) per million in the inoculum, since infectious dose was not identical for all donor samples. Lines indicate bacterial counts in tissues from five individual donors. [file image_1.png]

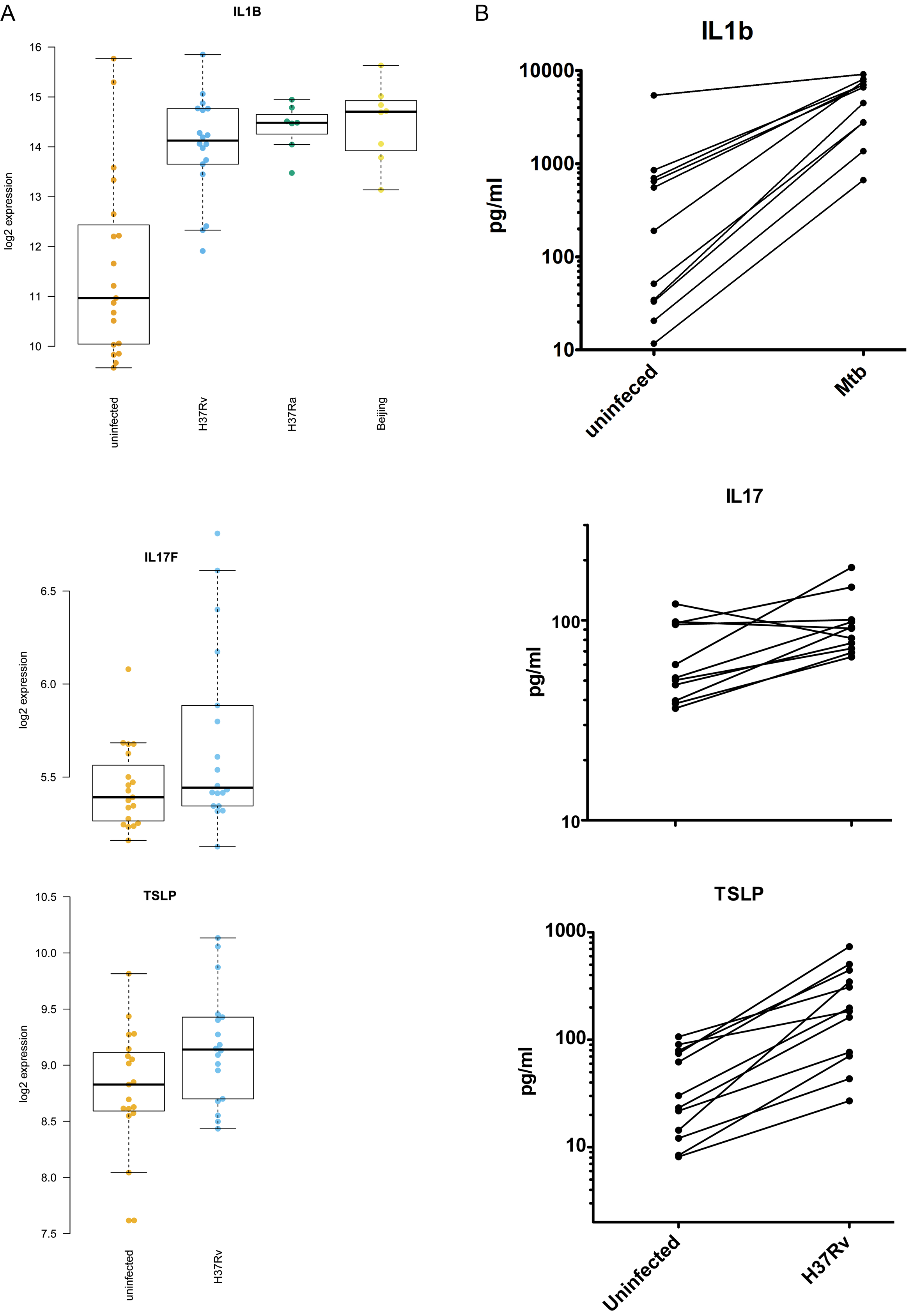

Supplement: Figure S2 — Induction of IL1B, IL17, and thymic stromal lymphopoietin (TSLP) by human lung tissue upon infection with Mycobacterium tuberculosis (Mtb). (A) Normalized log2 gene expression values in uninfected and Mtb infected tissue sections. Boxplots show median plus first and third quartile; whiskers extend to the most outer data point within 1.5 times the interquartile range. (B) Cytokine levels in supernatants from uninfected and Mtb (H37Rv) infected tissue sections. Cytokine levels are mean values from three individual tissue sections per donor with lines connecting values from uninfected and infected samples from each donor. [file image_2.png]

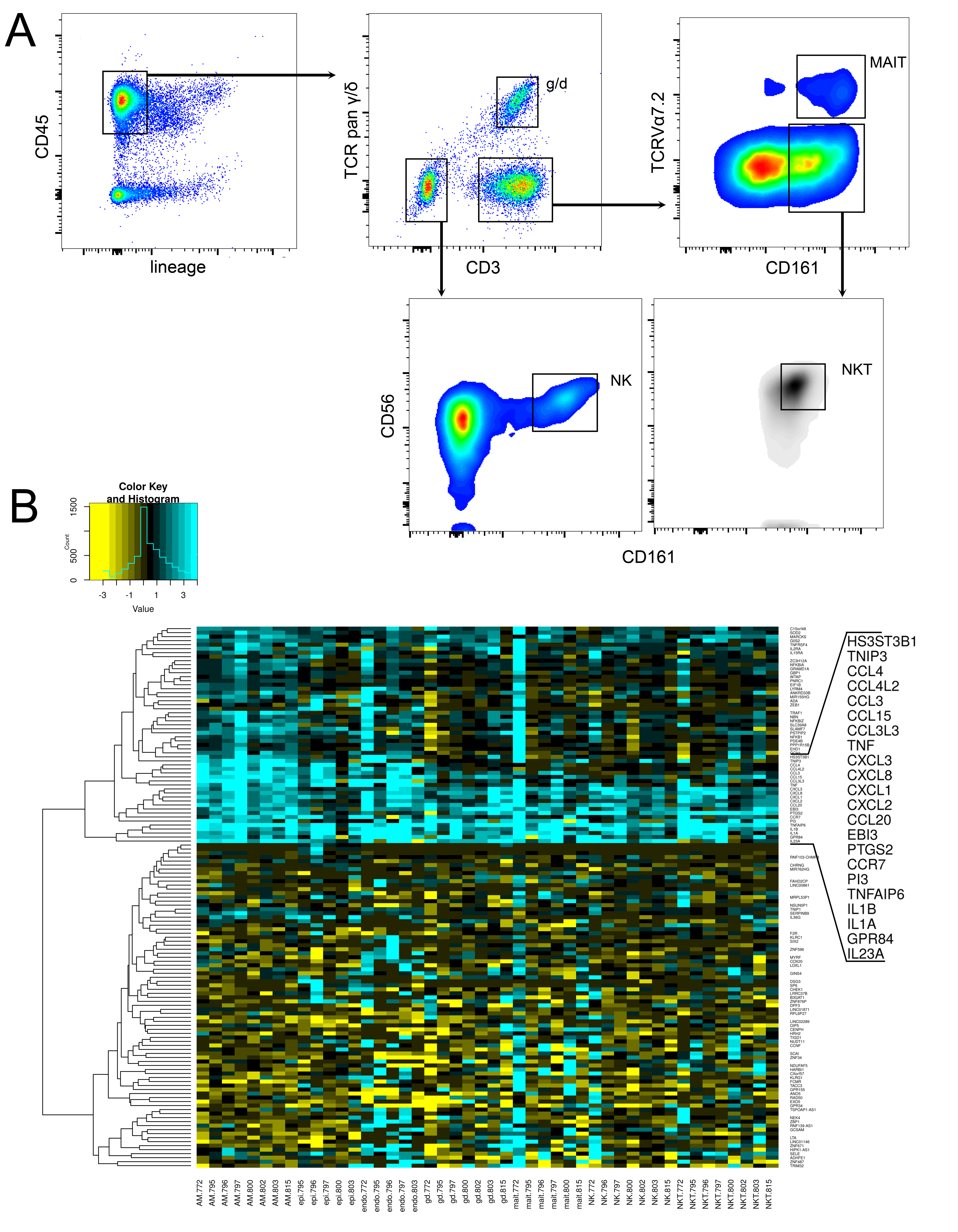

Supplement: Figure S3 — (A) Gating strategy for innate tissue-resident cells, gated on lymphocytes in forward-side scatter. (B) Heatmap showing log2 fold changes of top differentially expressed genes for each donor and in all analyzed innate cells. Genes shown are unique transcripts from a combined list of the top 50 differentially expressed genes in each cell type. [file image_3.png]

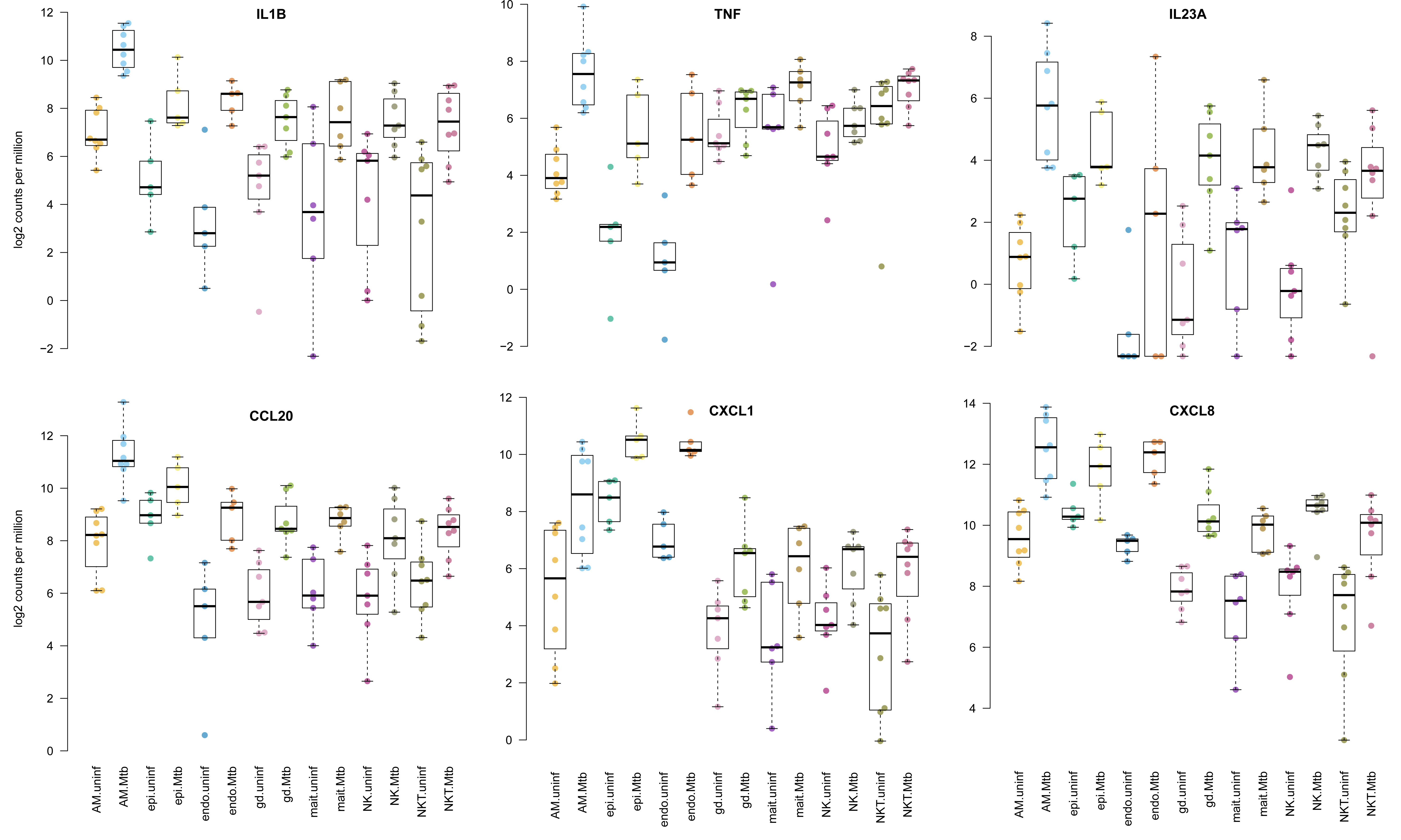

Supplement: Figure S4 — Expression and induction of proinflammatory chemokine genes upon Mycobacterium tuberculosis infection of human lung tissue. Plotted are log2 read counts per million sequenced reads from isolated cells for each donor tissue sample. Boxplots show median plus first and third quartile; whiskers extend to the most outer data point within 1.5 times the interquartile range. Each dot represents expression levels from an individual donor. [file image_4.png]

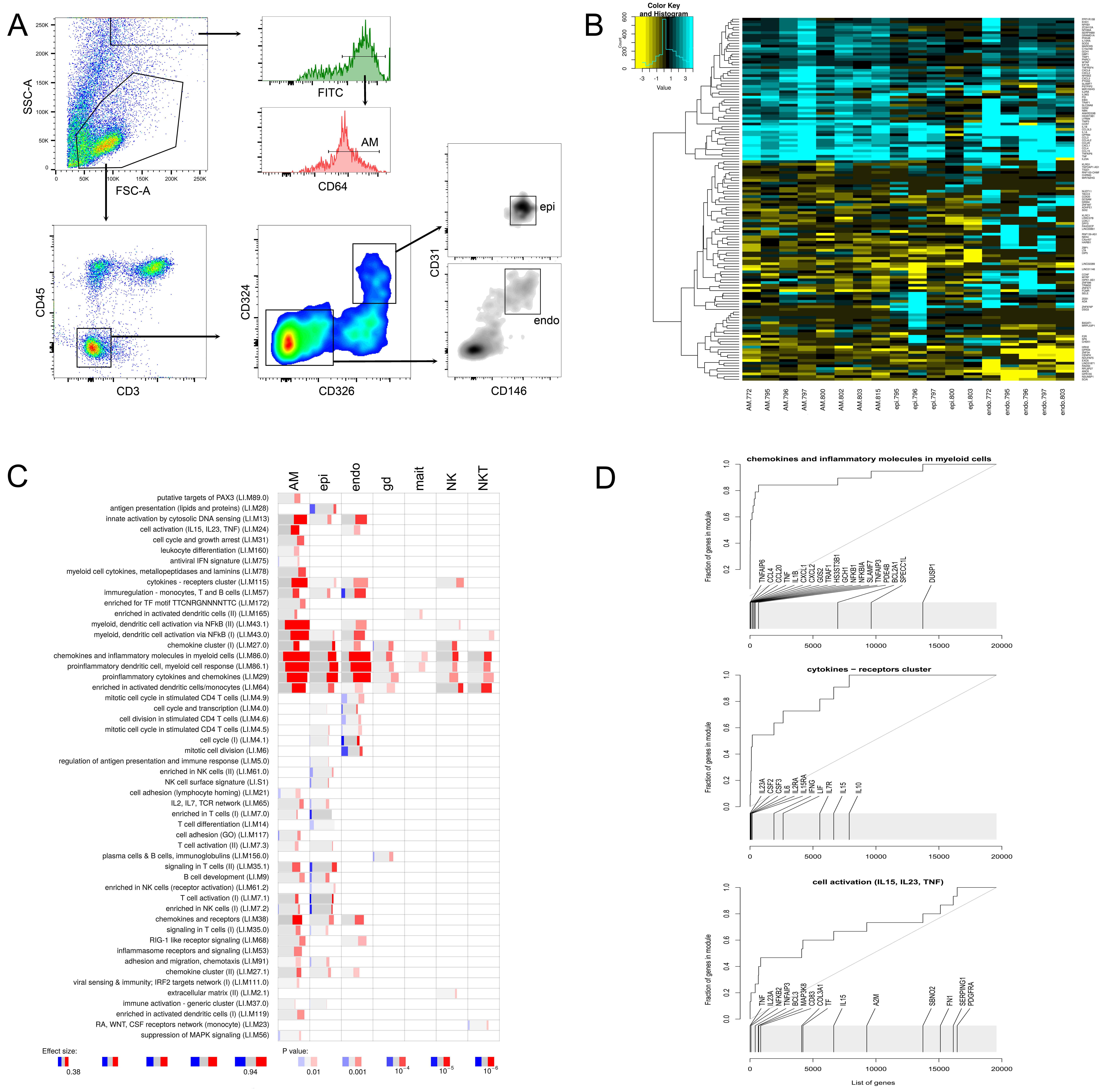

Supplement: Figure S5 — High resolution version of Figure 3. [file image_5.png]
